# Clinical and Pathologic Factors Affecting Lymph Node Yields in Colorectal Cancer

**DOI:** 10.1371/journal.pone.0068526

**Published:** 2013-07-16

**Authors:** Ta-Wen Hsu, Hsin-Ju Lu, Chang-Kuo Wei, Wen-Yao Yin, Chun-Ming Chang, Wen-Yen Chiou, Moon-Sing Lee, Hon-Yi Lin, Yu-Chieh Su, Shih-Kai Hung

**Affiliations:** 1 Department of General Surgery, Buddhist Dalin Tzu Chi General Hospital, Chiayi, Taiwan; 2 Teaching Division, Buddhist Dalin Tzu Chi General Hospital, Chiayi, Taiwan; 3 Department of Radiation Oncology, Buddhist Dalin Tzu Chi General Hospital, Chiayi, Taiwan; 4 School of Medicine, Tzu Chi University, Hualian, Taiwan; 5 Department of Hematological Oncology, Buddhist Dalin Tzu Chi General Hospital, Chiayi, Taiwan; Health Canada and University of Ottawa, Canada

## Abstract

**Objective:**

Lymph node yield is recommended as a benchmark of quality care in colorectal cancer. The objective of this study was to evaluate the impact of various factors upon lymph node yield and to identify independent factors associated with lymph node harvest.

**Materials and Methods:**

The records of 162 patients with Stage I to Stage III colorectal cancers seen in one institution were reviewed. These patients underwent radical surgery as definitive therapy; high-risk patients then received adjuvant treatment. Pathologic and demographic data were recorded and analyzed. The subgroup analysis of lymph node yields was determined using a *t*-test and analysis of variants. Linear regression model and multivariable analysis were used to perform potential confounding and predicting variables.

**Results:**

Five variables had significant association with lymph node yield after adjustment for other factors in a multiple linear regression model. These variables were: tumor size, surgical method, specimen length, and individual surgeon and pathologist. The model with these five significant variables interpreted 44.4% of the variation.

**Conclusions:**

Patients, tumor characteristics and surgical variables all influence the number of lymph nodes retrieved. Physicians are the main gatekeepers. Adequate training and optimized guidelines could greatly improve the quality of lymph node yields.

## Introduction

Colorectal cancer is one of the most frequently diagnosed cancers and a major cause of cancer deaths in Taiwan. [1] The primary treatment is resection, and adjuvant treatment is needed in high-risk patients. [2] Adjuvant treatment depends upon accurate staging. However, lymph node involvement is a highly important prognostic factor in colorectal cancer staging. [3] The results of several studies have been used to define the minimum number of lymph nodes to be harvested for accurate staging. Berberoglu et al. reviewed T1-4N0M0 colorectal cancer patients and reported that when more than 11 lymph nodes are examined, staging is more accurate. [4] In 2007, the National Quality Forum endorsed the harvest of 12 lymph nodes as a standard quality indicator for colorectal cancer resection specimens. [5] Therefore, lymph node yield during colorectal cancer surgery is being recommended as a benchmark of quality care. Recently, Chang et al. used propensity scoring to examine the number of lymph nodes harvested, and approved the number of lymph nodes as an indicator of quality of care. [6] Chung et al. analyzed the core measures and indicated these can be developed systematically and applied to improve quality of care. [7] However, despite these suggestions and guidelines, only 52% to 78% of hospitals comply. [8] Therefore, the objective of our study is to evaluate the impact of various factors upon, and identify independent associations with, lymph node harvest.

## Materials and Methods

### Ethics Statement

The study protocol was approved by the Buddhist Dalin Tzu Chi General Hospital Institutional Review Board (B10102003). The institutional review board waived the need for written informed consent from the participants because the data released from the hospital database were analyzed anonymously.

### Patient Population

The records of 166 patients with Stage I to Stage III colorectal cancer (TNM system) seen at one institution from August 2008 to June 2012 were reviewed. These patients received radical surgery as definitive therapy, and high-risk patients had adjuvant treatment. “High-risk factors” were defined as T3 or T4 lymph node involvement, or positive margins. All surgery was performed by colorectal specialists. Pathology data were obtained within 2 weeks after surgery. Pathology reports were reviewed to establish tumor size, grade, type, surgical margins, lymph nodes involved, perineural invasion, vascular permeation, lymphatic permeation, extracapsular spread (ECS), and specimen length. Other demographic data were recorded from electronic medical records and released from the hospital database. Four patients were excluded from the analysis because of loss to follow up (3 patients) or occurrence of a synchronous second primary (1 patient). All patients had colorectal cancer diagnosed histologically by pathologists and none had a prior history of cancer. All patients were informed about their disease treatment, including potential benefits and possible side effects, and were treated by multidisciplinary teams of colorectal surgeons, radiation oncologists, medical oncologists, and dieticians.

### Statistical Analysis

The subgroup analysis of lymph node yields was determined using a t-test and analysis of variants (ANOVA). In addition, a linear regression model and multivariable analysis were used to perform potential confounding and predicting variables. The appropriateness of the regression model was assessed by using influence-diagnostics-based leverage, studentized residuals and Cook’s distance. The model was then rerun without advanced deviation values. [9] SPSS 12.0 software (SPSS Inc, Chicago, IL, USA) was used for the analysis of all data. A statistically significant difference was defined as *P*<0.05.

## Results

**Figure 1:**
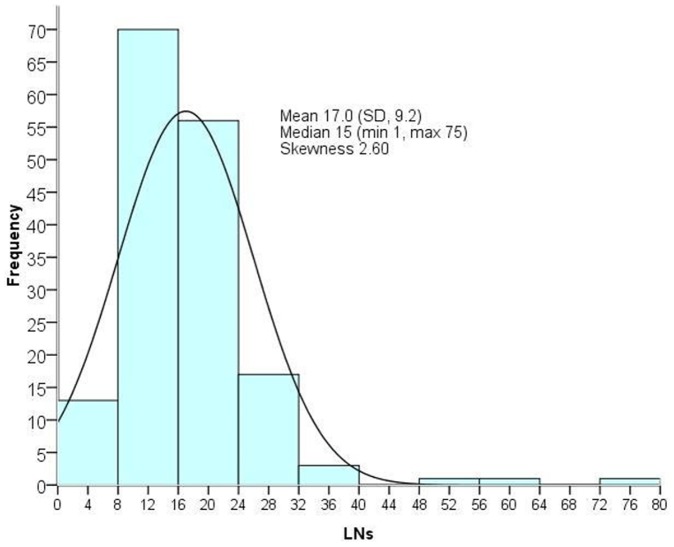
The distribution of lymph node count.

Patient characteristics are presented in Table 1. The mean age was 65.6 years (range: 28 to 90 years). When patients were grouped by diagnosis, there were 36, 70, and 56 patients in Stage I, Stage II and Stage III, respectively. More than half of the patients had laparoscopic resections. The mean specimen length was 17.86 cm (range: 6.7 to49.5 cm). In separate univariate analysis, tumor location, tumor size (dichotomized at 4 cm), specimen length (dichotomized at 13 cm), and the individual surgeon and pathologist had significant associations with lymph node yields (Table 2). The distribution of lymph node count was approximately symmetrical and close to a normal distribution (Figure 1). The mean number of lymph nodes was 17, and the median was 15. These variables were then analyzed using a multiple linear regression model (Table 3). After adjustment of other factors, five variables were significantly associated with lymph node yield. These were: tumor size, surgical method, specimen length, and surgeon and pathologist. The adjusted coefficient of determination (*R^2^*) was calculated to be 52.5% and 8.1% after removing significant variables. The explained value was 44.4% using these five significant variables.

**Table 1:** Descriptive statistics of patients, tumors and operative factors (n = 162).

Variables of interest	Mean or percent	Minimum-Maximum
Patient factors		
Age (yr)	65.57	28–90
Female gender	44%	
Body mass index (kg/m^2^)	24.06	15.1–33.7
Tumor factors		
T3/4	75%	
Node-positive	35%	
Poorly differentiated	12%	
Extracapsular spreading of lymph node	15%	
Lymphatic permeation	59%	
Vascular permeation	12%	
Neural invasion	20%	
Proximal margin positive	0%	
Distal margin positive	1%	
Peritoneum positive	12%	
Tumor size (cm)	4.48	0.4–15.0
Location in colon	47%	
Operative factors		
Laparoscopy	61%	
Specimen length (cm)	17.86	6.7–49.5

**Table 2:** Lymph node yields by subgroup.

Variable	n	Lymph nodes (mean)	SD	*P-*value
Patient factors				
Age (yr)				
≦ 65	74	17.2	9.7	0.74
>65	88	16.7	8.8	
Gender				
male	90	16.7	8.3	0.69
female	72	17.3	10.3	
Body mass index (kg/m^2^)				
≦ 28	140	16.7	9.6	0.37
>28	22	18.6	5.7	
Tumor factors				
Tumor location				
Colon	76	18.5	9.1	0.04
Rectal	86	15.6	9.1	
pT				
1–2	40	15.1	11.6	0.13
3–4	122	17.6	8.2	
pN				
0	106	16.6	10.6	0.56
1–2	56	17.5	5.6	
pStage				
I	36	15.4	12.2	0.52
II	70	17.3	9.8	
III	56	17.5	5.6	
Differentiated				
Well/moderately	143	17.1	9.5	0.47
Poorly	19	15.5	6.4	
Extracapsular spread of lymph node				
Negative	138	16.8	9.8	0.72
Positive	24	17.6	4.9	
Lymphatic permeation				
Negative	66	16.7	9.3	0.80
Positive	96	17.1	9.2	
Vascular permeation				
Negative	142	16.8	9.6	0.59
Positive	20	18.0	5.2	
Neural invasion				
Negative	129	16.2	7.4	0.15
Positive	33	19.9	14.0	
Peritoneum				
Negative	143	16.6	9.4	0.18
Positive	19	19.6	7.4	
Tumor size (cm)				
≦ 4	79	13.9	5.9	<0.05*
>4	83	19.9	10.7	
Operative factors				
OP method				
Laparoscopic resection	99	16.1	8.3	0.14
Open resection	63	18.4	10.4	
Specimen length (cm)				
≦ 13	46	14.1	5.6	0.01*
>13	116	18.1	10.0	
Surgeon				
A	13	17.6	7.0	0.03*
B	27	21.7	12.7	
C	122	15.8	8.1	
Pathologist				
A	35	16.9	7.5	<0.05*
B	37	26.0	4.4	
C	57	15.8	6.7	
D	33	13.6	5.4	

* Statistically significant difference (*P*<0.05).

**Table 3:** Linear regression model of lymph node yield.

	Variables of interest	Measure of effect	Standard error	*P-*value
Patient factors				
	Age (yr)	−0.54	0.04	0.15
	Gender	0.94	0.93	0.31
	Body mass index (kg/m^2^)	−0.05	0.13	0.73
Tumor factors				
	T-stage	0.79	0.88	0.37
	Total lymph node metastasis	0.29	0.22	0.18
	Differentiated	−0.04	1.35	0.98
	Extracapsular spread to lymph node	−1.12	1.73	0.52
	Lymphatic permeation	0.47	1.03	0.65
	Vascular permeation	−0.29	1.38	0.83
	Neural invasion	−1.60	1.40	0.26
	Peritoneum	2.31	1.79	0.20
	Tumor size (cm)	1.25	0.28	<0.05*
	Tumor location	−1.38	0.94	0.14
Operative factors				
	Surgical method	−2.95	1.23	0.02*
	Specimen length	0.17	0.08	0.03*
	Surgeon	3.54	1.64	<0.05*
	Pathologist	4.91	2.05	<0.05*

* Statistically significant difference (*P*<0.05).

## Discussion

The most important issue in a postoperative setting is accurate staging, particularly nodal staging. Survival is associated with an increasing number of lymph nodes analyzed. [10] In addition, the number of involved lymph nodes has been reported to be positively correlated with the number of lymph nodes examined. [11].

How to obtain adequate lymph nodes remains an important issue in colorectal cancer. In our study, we found that tumor size, surgical method, specimen length, and the individual surgeon and pathologist were significantly associated with lymph node yields.

Surgical variables influence the number of lymph node retrieved. The most important issue is the behavior of surgeons. Nicholl et al. reported that mean nodal yield increased among fellowship-trained surgeons and non-fellowship-trained surgeons alike. [12] The results of another study also demonstrated that advanced fellowship training of surgeons could increase lymph node yield. [13] In our study, the surgical approach and the individual surgeon were significantly associated with lymph node yields. However, the surgical method is dependent upon surgeon ability. Although patients and tumor characteristics are important factors in lymph node yields, they are only partly responsible for the variation. Quality of surgical resection may explain the variance proportion. Adequate training, to improve lymph node harvest, could improve the quality of cancer care.

Variances among pathologists were as striking as those among surgeons. When Mekenkamp et al. selected patients from a multicenter prospective randomized trial, they found great variations between pathology laboratories and individual pathologists. [14] Some studies reported the importance of the use of fat solvents or optimal specimen fixation. [15], [16] Pathologists should have a standardized pathology procedure for lymph node harvesting. Storli et al. used a questionnaire to examine ways different institutions handled colon cancer specimens and found variations in the routines of each department. [15] However, variations existed even within a single institution. A standardized pathology guideline is essential because optimal tissue handling can improve assessment outcome.

Tumor characteristics also influenced lymph node yields. Larger tumors may be more visible on pathologic examination due to increased cancer antigen and inflammation response. [17] Chou et al showed that with every 1-cm increase in tumor size, there was an average increase of 2% to 3% in the number of lymph nodes examined in colorectal cancer specimens. [5] Nash et al. observed an increase in average lymph nodes yields among T3/T4 tumors versus T0–T2 tumors. [9] In our study, tumors larger than 4 cm had more lymph node yields than did smaller tumors. In addition, tumor size has been shown to be a more important predictor of lymph node yield than is tumor stage. [9].

The results of our study also indicated that the other positive predictor of lymph node retrieval, after multivariate analysis, was specimen length. Specimen length correlated positively with the number of nodes retrieved, by linear regression analysis, and the dichotomized length which regarded lymph node yields was 13 cm. Similar to our results, Norwood et al. demonstrated that a longer specimen length results in a higher lymph node retrieval rate. [18] In another study, the anatomic site of the tumor influenced the number of lymph node examination and decreased lymph node retrieval with distal tumor. [19] This may help to explain why longer anatomical specimens, such as those from an extended right hemicolectomy, have relatively higher nodal yields. [18] Interestingly, Nash et al. further demonstrated that measuring the extent of mesenteric resection using the number of vascular pedicles was a significant predictor of lymph node yield, independent of the length of bowel resected. [9].

In our study, five variables were significantly associated with lymph node yield. They were tumor size, surgical method, specimen length, and surgeon and pathologist. From literature reviews, factors significantly affecting the lymph node yields among different hospitals may be different. [20] For improving lymph node yield in our hospital, we try to propose strategies. The efforts included regular cancer treatment combine meeting that surgeons and pathologists could mutual communicate. Furthermore, the consensus of adequate training for surgical quality and a standardized procedure for specimen handled were also obtained. These methods could help to improve cancer care. We also suggest that every hospital should review own results to adhere a benchmark of quality care and find out the factors that can be improved by specific strategies.

### Conclusions

Patients, tumor characteristics and surgical variables influence the number of lymph nodes retrieved. Physicians are the main gatekeepers. Adequate training and optimized guidelines could greatly improve the quality of lymph node yields.
